# PYK-SubstitutionOME: an integrated database containing allosteric coupling, ligand affinity and mutational, structural, pathological, bioinformatic and computational information about pyruvate kinase isozymes

**DOI:** 10.1093/database/baad030

**Published:** 2023-05-03

**Authors:** Liskin Swint-Kruse, Larissa L Dougherty, Braelyn Page, Tiffany Wu, Pierce T O’Neil, Charulata B Prasannan, Cody Timmons, Qingling Tang, Daniel J Parente, Shwetha Sreenivasan, Todd Holyoak, Aron W Fenton

**Affiliations:** Department of Biochemistry and Molecular Biology, The University of Kansas Medical Center, 3901 Rainbow Blvd., Kansas City, KS 66160, USA; Department of Biochemistry and Molecular Biology, The University of Kansas Medical Center, 3901 Rainbow Blvd., Kansas City, KS 66160, USA; Department of Biochemistry and Molecular Biology, The University of Kansas Medical Center, 3901 Rainbow Blvd., Kansas City, KS 66160, USA; Department of Biochemistry and Molecular Biology, The University of Kansas Medical Center, 3901 Rainbow Blvd., Kansas City, KS 66160, USA; Department of Biochemistry and Molecular Biology, The University of Kansas Medical Center, 3901 Rainbow Blvd., Kansas City, KS 66160, USA; Department of Biochemistry and Molecular Biology, The University of Kansas Medical Center, 3901 Rainbow Blvd., Kansas City, KS 66160, USA; Chemistry Department, Southwestern Oklahoma State University, 100 Campus Dr., Weatherford, OK 73096, USA; Department of Biochemistry and Molecular Biology, The University of Kansas Medical Center, 3901 Rainbow Blvd., Kansas City, KS 66160, USA; Department of Biochemistry and Molecular Biology, The University of Kansas Medical Center, 3901 Rainbow Blvd., Kansas City, KS 66160, USA; Department of Family Medicine and Community Health, The University of Kansas Medical Center, 3901 Rainbow Blvd., Kansas City, KS 66160, USA; Department of Biochemistry and Molecular Biology, The University of Kansas Medical Center, 3901 Rainbow Blvd., Kansas City, KS 66160, USA; Department of Biochemistry and Molecular Biology, The University of Kansas Medical Center, 3901 Rainbow Blvd., Kansas City, KS 66160, USA; Department of Biology, University of Waterloo, 200 University Ave. W, Waterloo, ON N2L 3G1, Canada; Department of Biochemistry and Molecular Biology, The University of Kansas Medical Center, 3901 Rainbow Blvd., Kansas City, KS 66160, USA

## Abstract

Interpreting changes in patient genomes, understanding how viruses evolve and engineering novel protein function all depend on accurately predicting the functional outcomes that arise from amino acid substitutions. To that end, the development of first-generation prediction algorithms was guided by historic experimental datasets. However, these datasets were heavily biased toward substitutions at positions that have not changed much throughout evolution (i.e. conserved). Although newer datasets include substitutions at positions that span a range of evolutionary conservation scores, these data are largely derived from assays that agglomerate multiple aspects of function. To facilitate predictions from the foundational chemical properties of proteins, large substitution databases with biochemical characterizations of function are needed. We report here a database derived from mutational, biochemical, bioinformatic, structural, pathological and computational studies of a highly studied protein family—pyruvate kinase (PYK). A centerpiece of this database is the biochemical characterization—including quantitative evaluation of allosteric regulation—of the changes that accompany substitutions at positions that sample the full conservation range observed in the PYK family. We have used these data to facilitate critical advances in the foundational studies of allosteric regulation and protein evolution and as rigorous benchmarks for testing protein predictions. We trust that the collected dataset will be useful for the broader scientific community in the further development of prediction algorithms.

**Database URL** https://github.com/djparente/PYK-DB

## Introduction

To identify important differences among human genomes, to understand the impact from changes that occur as viruses evolve and to engineer novel protein function, scientists need improved predictions about the functional outcomes that result from amino acid substitutions. To that end, numerous algorithms have been developed. The first-generation prediction algorithms incorporated assumptions derived from and tested upon mutational datasets that were generated over several decades of research. However, most of these historical substitution studies were inadvertently biased to positions that have been conserved throughout evolution. This bias precludes accurate predictions for substitutions at other types of positions: in particular, predictions are poor for a special class of positions—which we have defined as ‘rheostat’ positions (1)—that do ‘not’ follow the rules that are commonly assumed for substitutions at conserved positions (Table 1) (1–10).

**Table 1.** Common assumptions about substitution rules fail for some amino acid positions

Assumptions from historic studies	Refutation
At ‘important’ protein positions ….	At ‘rheostat positions’ ….
1. Most amino acid substitutions are highly detrimental to structure or function.	The 20 possible substitutions lead to a wide range of functional outcomes. Thus, at a single rheostat position, only a few substitutions are highly detrimental, whereas most exhibit intermediate outcomes and some exhibit enhancing outcomes.
2. Chemically similar side chains can substitute for each other without much detriment.	Chemistries of the substituted amino acids often have little correlation with their functional outcomes. In other words, chemically dissimilar side chains can have more similar functional outcomes than do chemically similar side chains.
3. Substitution outcomes can be extrapolated among homologs.	Functional outcomes from substitutions do ‘not’ extrapolate among homologs.

More recently, positions with a wider range of conservation (and non-conservation) have been assessed in ‘deep mutational scanning’ experiments (11–14). However, the functional readout for these studies is often indirect (e.g. biological survival) or of low resolution (e.g. cell sorting). Moreover, these readouts often agglomerate multiple functional parameters; as such, the outcomes associated with different substitutions could arise from different biochemical parameters. For example, mutation outcomes in an enzyme might arise from altered substrate binding, catalytic rate, product binding or allosteric regulation. Deep mutational scanning assays can also be sensitive to protein expression levels, although various strategies can help parse these changes from functional changes (1, 7, 8, 15). As such, although data from deep mutational scanning are useful for training prediction algorithms that are based on machine learning, they do not contain the information needed to predict functional change from fundamental chemical principles (16).

Therefore, our (6, 9, 10, 17–20) and others’ (21–23) approach to addressing the need for improved predictive algorithms has been to create large, biochemical datasets. The database described herein collates the wide range of biochemical, mutational, bioinformatic, structural, pathological and computational studies that have been performed for the homologs of the pyruvate kinase (PYK) isozyme family. The centerpiece of this database is the quantitative, biochemical characterization for more than 1000 variants. Another key feature is that many of the characterized positions were subjected to site-specific, semi-saturating mutagenesis (semiSM; 10–12 substitutions per position).

We reached this semiSM design after considering (and sometimes implementing) several other designs. For example, alanine and glycine scans have been very popular for ‘removing’ the side chain to determine the role of each position (19). However, an alanine variant may function similarly to the native protein even if the substituted position contributes to function (1, 24). Therefore, the functional contributions of such positions may be overlooked if other amino acid substitutions are excluded (24). Furthermore, if only one amino acid substitution is chosen, results cannot discriminate whether the original amino acid was required for function (a loss-of-function mutation) or the substituted amino acid was simply not tolerated (a negative gain-of-function mutation) (5, 10, 24–26).

These results demonstrate that multiple amino acid substitutions must be assessed for each position. Ideally, 19 substitutions would be created at each position *via* site-saturating mutagenesis (SSM), but this approach can be prohibitively expensive. One approach for simplifying experimental design is to use representative amino acids from chemically similar groups. Unfortunately, this has led to additional bias: as mentioned in Table 1, chemically similar amino acids do not always have similar substitution outcomes at non-conserved positions (1, 3–5, 7, 9, 10, 25, 26).

Fortunately, we have found that the overall functional role of a ‘position’—rather than of a ‘residue’—can be determined from just 10–12 amino acid substitutions (‘semiSM’) (5). To describe each position’s functional role, we previously devised the positional classification system of ‘toggle’, ‘neutral’ and ‘rheostat’ positions (5) that is reviewed in Supplemental Information 1. In brief, (i) at toggle positions, most substitutions abolish function (‘dead’) (2); (ii) at neutral positions, most substitutions function like the wild type (6) and (iii) at rheostat positions, each substitution leads to a different effect on the measured functional parameter, and the set of substitutions samples at least half of the parameter’s accessible range (as defined by wild type and dead) (1, 5). This change of perspective—from the effects of individual substitutions to the overall roles of positions—allows biochemical semiSM results to be readily correlated with bioinformatic and structural data and compared among homologs.

Indeed, the widespread existence of rheostat positions—which do not follow the expected substitution rules (Table 1)—mandates biochemical and biophysical SSM/semiSM studies in order to understand how each amino acid position contributes to a protein’s biochemical properties. Although the first rheostat positions were identified in studies of non-conserved positions (1), later studies showed that properties derived from sequence alignments only partially discriminate rheostat positions from neutral or toggle positions (9, 17). Furthermore, no striking structural changes have been associated with the functional outcomes from substitutions at rheostat positions (8, 18). Thus, more examples must be collected. The current database includes multiple examples of human liver pyruvate kinase (hLPYK) rheostat positions, along with their structural and bioinformatic characteristics.

## Overview of PYK function

PYK catalyzes the last step in glycolysis and is present in all domains of life. Humans have four PYK isozymes. hLPYK (expressed in the liver) and hRPYK (expressed in mature erythrocytes) are two products of the *PKLR* (*l/r-pyk*) gene that are generated from alternative start sites. hM_1_PYK (PKM1; expressed in the skeletal muscle, heart and brain) and hM_2_PYK (PKM2; expressed in all fetal tissues, adult smooth muscle and many cancers) are two gene products from the *PKM* gene that are generated *via* alternative splicing. Among these, hLPYK controls liver homeostasis between glycolysis and gluconeogenesis to regulate blood glucose levels. In contrast, the PYK isozyme expressed in the skeletal muscle (M_1_PYK) appears to have less control over the glycolytic pathway. These and other features of the human isozymes have been extensively reviewed (27–30).

Consistent with their tissue-specific regulatory roles, PYK isozymes have evolved a range of allosteric and post-translational modifications that regulate their enzymatic activities. The predominant regulation in most known PYK isozymes is *via* binding to phosphorylated sugars that allosterically improve affinity (*K_app_*) for the substrate phosphoenolpyruvate (‘PEP’). For the flagship isozyme used in many of our studies, hLPYK, affinity for PEP is improved when the allosteric activator fructose-1,6-bisphosphate (abbreviated as ‘Fru-1,6-BP’ in text, ‘FBP’ in subscripts and figures and ‘F16BP’ in the database) is bound; in addition, PEP affinity is reduced when the allosteric inhibitor alanine is bound at yet another allosteric site. Several other PYK isozymes exhibit both activating and inhibiting allosteric regulation. In contrast, a few isozymes, such as *Zymomonas mobilis* PYK (‘ZmPYK’), are ‘not’ allosterically regulated (18, 31–33).

**Figure 1.**
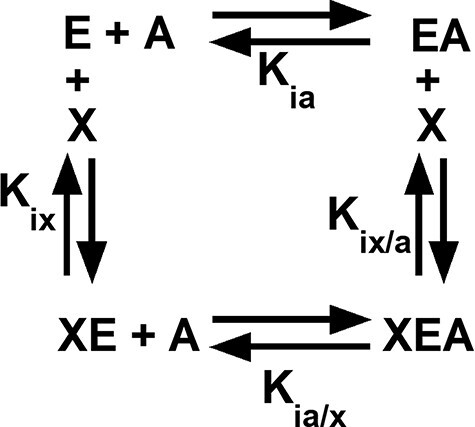
An allosteric energy cycle in which an enzyme (*E*) binds one substrate (*A*) and one allosteric effector (*X*). *K_ia_* is the binding of the substrate to the enzyme in the absence of the effector; when initial data are derived from kinetic measurements, this parameter is designated as *K_a_*. *K_ia/x_* is the binding of the substrate to the enzyme in the presence of saturating concentrations of the effector. *K_ix_* is the binding of the effector to the enzyme when the substrate is absent. *K_ix/a_* is the binding of the effector to the enzyme in the presence of saturating concentrations of the substrate. Allosteric coupling is defined as *Q_ax_* = *K_a_/K_a/x_* = *K_ix_/K_ix/a_* (34, 36, 37, 223). A description of hLPYK’s allosteric regulation requires two such functional cycles—one for activation by Fru-1,6-BP and one for inhibition by alanine.


Among the regulated PYK, both allosteric inhibition and activation can be defined by an allosteric energy cycle (Figure 1) (34–38). When characterizing two allosteric functions, as for hLPYK, five functional parameters are quantified (Figure 1): (i) *K_a-PEP_* (the kinetic equivalent to *K_ia-PEP_*), the apparent affinity for PEP; (ii) *K_ix-Ala_*, the binding of Ala; (iii) *K_ix-FBP_*, the binding of Fru-1,6-BP; (iv) *Q_ax-Ala_*, the allosteric coupling constant that relates PEP and Ala binding to the protein, and (v) *Q_ax-FBP_*, the allosteric coupling constant that relates PEP and Fru-1,6-BP binding to the protein. Mathematically, *Q_ax_* is defined as follows:

(1) $${Q_{ax}} = {{{K_a}} \mathord{\left/
{\vphantom {{{K_a}} {{K_{a/x}}}}} \right.
-} {{K_{a/x}}}} = {{{K_{ix}}} \mathord{\left/
{\vphantom {{{K_{ix}}} {{K_{ix/a}}}}} \right.
-} {{K_{ix/a}}}},$$

where *K_a_* is the kinetically derived apparent affinity of the enzyme for PEP in the absence of the effector and *K_a/x_* is the affinity of the enzyme for PEP when the effector is present at saturating concentrations. *K_ix_* is the binding affinity for the effector in the absence of PEP, and *K_ix/a_* is the binding affinity of the effector in the saturating presence of PEP. Note that the effector binding and allosteric coupling constants need not be correlated; indeed, many substitutions at rheostat positions do not show correlated functional changes in those parameters (10).

A key feature of these experiments is that ligand affinity is evaluated in an enzyme concentration–independent measurement. That is, *K_a_* can be determined from ½ *V_max_* without knowing the enzyme concentration. Since allosteric coupling is a ratio of ligand binding values, *Q* can also be evaluated without knowing the enzyme concentration. This allows for the quantitative evaluation of substrate affinity, allosteric effector binding and allosteric coupling to be completed in partially purified protein samples, thereby facilitating the throughput for a semiSM study. Importantly, the independence from enzyme concentration also ensures that functional data are not ‘contaminated’ with effects on protein stability and provide ‘pure’ information about functional changes. (A limitation is that effects on *k_cat_* are not determined in this assay.) Examination of the variants collected in this database illustrate that all five of these functional parameters can be affected by substitutions in hLPYK, with outcomes that range over orders of magnitude (3, 5, 9, 10).

## Overview of PYK structure

**Figure 2.**
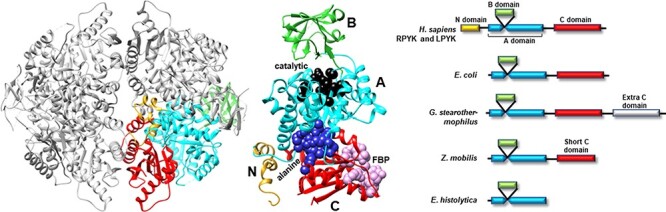
Representative PYK structures. Left: the homotetramer of hLPYK (PDB4IMA); three monomers are in gray, and the fourth is colored by a domain. Middle: the ribbon of the hLPYK monomer is colored by domains (A, B, C and N). The catalytic site is in black space-filling, the inhibitory alanine binding site is in blue space-filling and the enhancing Fru-1,6-BP binding site is in pink space-filling. Right: alternative domain structures observed throughout the PYK family. Domain colors match those of the middle panel.

Many PYK isozymes are homotetramers (27–30) (Figure 2, left); a few PYK isozymes are homodimers (17, 31–33, 39). In most isozymes, each monomer comprises three domains (Figure 2, center). The PYK ‘A’ domain is a TIM barrel (40–44), the sequence of which is interrupted by the ‘B’ domain. The B domain appears to serve as a lid to the catalytic site, which is located on the top surface of a TIM barrel (45–51). The B domain is highly flexible (52) and adopts many conformations in the various known crystal structures. The third PYK domain (‘C’ domain) is located on the other side of the TIM barrel relative to the catalytic site and B domain. In many PYKs, the C domain can contain as many as three distinct allosteric sites (53–55). Isozymes from some species can either lack the C domain or have acquired an extra C domain (Figure 2, right) (33, 56). Isozymes similar to human liver (hLPYK) and erythrocyte (hRPYK) PYK have an extra N-terminal domain of varied length that includes a regulatory phosphorylation site, e.g. (57–59) and references therein.

## Database overview

In addition to summarizing the work carried out in our laboratory on hLPYK [(6, 9, 19) and previously unpublished data], rM_1_PYK (47–49, 52, 55, 60–64), ZmPYK (17) and other isozymes studied using mutagenesis, we have assembled a wide range of PYK data from the literature including sequences, structures, disease-causing substitutions, biochemical characterizations and computations (6, 9, 10, 25, 26, 50, 57, 58, 61, 62, 65–161). Data are presented in a format designed to facilitate their use in future efforts to advance the development of algorithms that predict substitution outcomes. The PYK-SubstitutionOME database comprises two main units, and an updateable format is maintained at https://github.com/djparente/PYK-DB.

Unit 1 comprises an Excel workbook, in which the first worksheet presents a summary data table and is supported by additional worksheets containing expanded data tables and links to other databases (e.g. BRENDA and GNOMAD) that contain up-to-date information on PYK. The supporting worksheets are formatted, so they can be exported as CSV files for use in various database programs. Throughout the database, data are associated with their original publication(s) using PubMed ID numbers (‘PMIDs’). These PMIDs are also cited in the relevant text below; additional publications cited in the Supplemental Information include references (162–170).

Unit 2 contains three PYK sequence alignments in FASTA format. Two of these were originally published in earlier studies (9, 17, 136), and these manuscripts provide the computational details of their construction. In brief, the ‘PYK Pendergrass 2006’ multiple sequence alignment (MSA) contains manually curated sequences from a BLAST (173) search that sampled the whole PYK family across all domains of life; this MSA was used to compute the various bioinformatic scores reported in Unit 1 of the database. *E*-values are exceedingly low across the MSA (BLAST (171)), even though sequence identities range as low as 18%. We and others who have performed sequence analyses of this family (172–174) have found that these homologs only comprise PYK isozymes; PYK function has been verified for multiple organisms by extensive studies throughout the 1960s—1980s; many of these studies are collected from the BRENDA database [(170); https://www.brenda-enzymes.org/enzyme.php?ecno=2.7.1.40]; no known homologs lack the canonical PYK catalytic activity.

The second MSA (‘hLPYK subfamily 2022’) was generated to check that the 2006 sampling of the whole family produced comparable results (17) (i.e. the sequences deposited in 2006 provided good representation of the sequences available in 2022). Finally, using new tools developed for large datasets in the past year, we include a new, 2000-sequence MSA for the whole family that was generated in ConSurf (176) using an HMMR search (177) of their ‘Clean UniProt’ database (‘PYK whole family 2023 CleanUniProt.fas’). (As of this writing, UniProt contains >60,000 PYK sequences.) The comparison of ConSurf scores for the 2006 and 2023 alignments yields a Pearson correlation coefficient of 0.80 and a Spearman correlation coefficient >0.95; most of the variation occurs at the least conserved positions or the C terminus, again verifying the continued usefulness of the PYK Pendergrass 2006 MSA. (We do note that the ConSurf default value of 150 sequences provided insufficient sampling of the huge PYK family, which was determined by assessing progressively larger MSAs, until ConSurf scores converged.)

## Sections of the PYK-SubstitutionOME workbook (Unit 1)

In the PYK-SubstitutionOME workbook, the first worksheet (‘Features of PYK Positions’) provides an overview of the whole database and summary details for each amino acid position. This main table is formatted, so that the information in Columns C and D introduces the various features (rows) and Column E through VT provide information about this feature for each PYK amino acid position. The left-most columns (A and B) contain legends to aid the interpretation of various scores. The second worksheet in this workbook, ‘Legends and Definitions’, contains brief descriptions of the data, definitions and expanded color legends for each of the four major sections. As further described, each row contains different types of information about the PYK positions, some of which are expanded in the supporting worksheets.

### Sequences and common structural features

**Figure 3.**
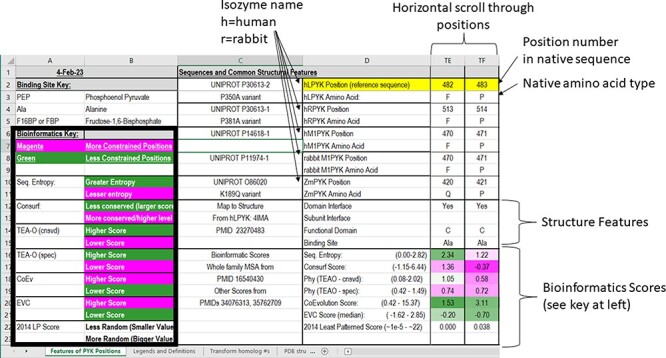
The top two sections of the ‘Features of PYK Positions’ page from PYK-SubstitutionOME. The left-most rows contain reference information and abbreviations. The top rows (1–11) contain the position numbers and wild-type amino acids for hLPYK, hRPYK, hM1PYK, rM1PYK and ZmPYK. Note that Columns A–D are locked, allowing the position columns to be scrolled. The next section’s rows (12–23) include notes on the structural locations for each position and evolutionary scores derived from the MSAs in Unit 2 (9, 17, 136). Each evolutionary score type has a different range, which is indicated in parentheses by the score names. To aid interpretation, the scores for each position are colored to show where it falls in the range, with magenta indicating stronger constraint/more prevalent pattern and green indicating less (see the legend highlighted with the black box on the left of this figure) .

In the first section of the ‘Features of PYK Positions’ worksheet, the first set of rows contains the sequence numbers and wild-type amino acids for the main PYK isozymes that we have studied (Figure 3). The first two, hLPYK and hRPYK, are products from the same gene arising from alternative start sites. The other isozyme sequences in the main table are human muscle PYK (hM_1_PYK), rabbit muscle (rM_1_PYK; a historic model system; (178, 179)) and ZmPYK. ZmPYK was included because we have studied it as an example isozyme that has different properties: ZmPYK is dimeric rather than the standard tetramer, lacks allosteric regulation and has followed an isolated evolutionary path (17). The supporting worksheet ‘Transform Homolog #s’ contains a table that aligns and numbers the analogous positions in the human, *Z. mobilis* and several other homologs contained in this database; the right side of this worksheet (yellow cells) also contains a formula to readily transform the homolog amino acid position numbering system to that of a common reference sequence (herein, hLPYK). The Python code used to create this worksheet from sequence alignments (Unit 2) is presented in Supplemental Information 2.

The next set of rows in the first section of the main worksheet associates each position with its major structural details. These include domain locations, domain and subunit contacts and direct ligand/substrate contacts. Although based on an hLPYK structure (4IMA), we expect these features to be common among most PYK isozymes. An overview of all available structures and their citations is on the supporting worksheet ‘PDB Structures Available’ (as of October 2022), along with their source organism and bound ligands (40, 44, 46–49, 53–56, 58, 60, 64, 83, 132, 139, 146, 180–216). Information about the allosteric ligands documented for other isozymes can be found at the BRENDA database (175), the link for which is included on the ‘Other Resources’ worksheet in the PYK-SubstitutionOME workbook.

Below the structural information, the third set of rows in the first section of the main table contains a range of bioinformatic scores derived from analyses of the PYK MSA from Pendergrass 2006. The FASTA-formatted sequence alignment used to determine these scores (9, 136)—which contains additional PYK sequences not in the main table—is included as a separate text file in Unit 2. For the PYK subfamily that includes the four human isozymes, a recently expanded MSA [March 2022 (17)] is also included as a FASTA-formatted text file in Unit 2 (‘hLPYK subfamily 20022.fas’).

These different bioinformatic scores provide various ways to determine ‘non-conservation’ for each PYK position. The generation of the bioinformatic scores was previously described (9, 17). In brief, the ‘sequence entropy’ of a given position is the simplest measure of amino acid change throughout the protein family, quantifying and aggregating the frequency with which the 20 amino acids occur at that position in the alignment ($ - \mathop \sum \limits_{k = 1}^{20} {p_k}{\rm{*}}\log {p_k}$, where *p* is the observed frequency of amino acid side-chain *k* at a given amino acid position) (217). ConSurf (176) scores report evolutionary rates determined from both sequence entropy and the evolutionary relatedness derived from the branch lengths of the family’s phylogenetic tree. Scores are reported on a scale of 1 (least conserved) to 9 (most conserved ‘within’ the analyzed MSA). TEAO (210) analyses are similar to those of ConSurf but reported as two sets of scores: ‘conservation’ assesses how well a position is conserved across the whole family and ‘specificity’ assesses whether a position is non-conserved in the whole family but conserved within phylogenetic branches. Co-evolutionary analyses detect pairs of positions that change in concert (219), and eigenvector centrality (‘EVC’) analyses identify positions with multiple strong partners (163). The ‘least patterned’ score was used to identify positions that show the least pattern of change, which has been associated with positions that have neutral substitution phenotypes (see Supplemental Information 1) (6).

### Natural variants identified

**Figure 4.**
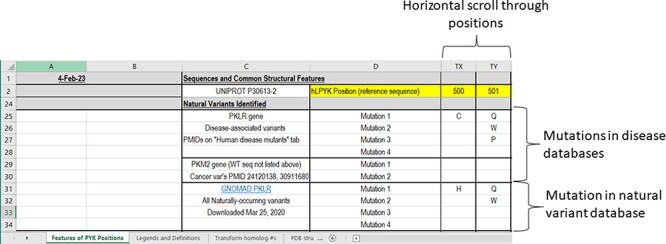
The third section of the ‘Features of PYK Positions’ worksheet in the PYK-SubstitutionOME workbook. Rows 1 and 2 are anchored to provide position reference points, as are Columns A–D. This third section contains substitutions associated with PKD and other substitutions identified in the human population; the latter are not necessarily associated with disease.

Progressing down the ‘Features of PYK Positions’ page, the second major section notes the naturally occurring hRPYK substitutions that have been associated with disease (Figure 4). Mutations in RPYK can lead to a pyruvate kinase deficiency (PKD) that results in non-spherocytic anemia. In fact, mutations in the hRPYK protein represent the second largest set of mutations in one protein that result in medically relevant enzymopathies (220). Citations for these disease mutants are in the supporting worksheet ‘Human Disease Mutants’. Although limited in number, several mutations in hM_2_PYK have been associated with cancer and are also listed in the main PYK-SubstitutionOME worksheet. The human isozymes also have other variants (not noted as disease causing) that have been observed in exome sequencing. For hRPYK, these were extracted from the GNOMAD database (221) in March 2020 for one of our studies (3) and are included in PYK-SubstitutionOME. Since the observed variants for all human isozymes are updated frequently within the GNOMAD database, we provide links to the user for the most up-to-date information (see the ‘Other PYK Data Resources’ supporting worksheet, which is the last worksheet of the PYK-SubstitutionOME workbook).

### *In vitro* biochemical characterization of PYK substitutions

**Figure 5.**
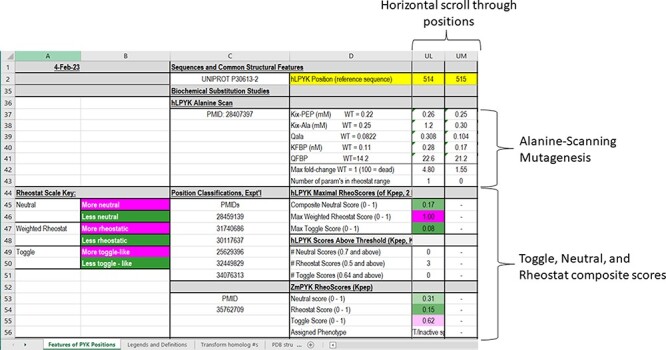
The fourth section of the ‘Features of PYK Positions’ worksheet in the PYK-SubstitutionOME workbook. Again, Rows 1 and 2 and Columns A–D are anchored to provide reference points; the K and Q values for wild-type hLPYK measured under the same experimental conditions (‘WT = [value]’) are included for reference. This section contains information from *in vitro* biochemical characterizations of purified [and partially purified (19)] PYK variants. Both information from a whole-protein, alanine-scanning mutagenesis study and RheoScale scores from semi-SM (i.e. toggle, neutral and rheostat scores) are entered here for each position. Each RheoScale score ranges from 0 to 1; the significant ranges are indicated in parentheses, and their corresponding color legends that aid visual interpretation are noted at left. Experimentally determined parameters for >1000 substituted hLPYK proteins are included in supporting worksheets.

The third major section in ‘Features of PYK Positions’ summarizes the most unique data of our database: the comprehensive biochemical data of more than 1000 PYK variants that we have collected over the past decades (Figure 5). Where available, data are included for five functional parameters routinely measured in our studies (e.g. Figure 1). We note that these parameters can be affected by a wide range of conditions—buffer, pH, counter-ions, other substrates used in coupled assays, etc. Thus, for each dataset, it is always important to compare variants to their wild-type counterpart to provide context for interpreting the change that accompanies any substitution.

Within this third section of the ‘Features of PYK Positions’ worksheet, the first set of rows summarizes the parameters measured in a whole-protein alanine-scanning substitution study of hLPYK (19). For reference, wild-type K and Q values measured under the same experimental conditions are included in Column D. To easily identify positions in the alanine-scanning data that alter any functional parameter, the ‘Max fold-change’ row reports the largest fold-change (i.e. the variant parameter divided by the equivalent wild-type parameter) observed among the five parameters; because this calculation was first developed for a study of neutral positions (6), fold-change enhancing and diminishing are reported the same (e.g. both 10-fold enhancing and 10-fold diminishing are reported as ‘10’). A few alanine variants lacked any enzymatic activity; these are denoted with ‘100’ in the ‘Max fold-change’ row, whereas columns corresponding to unsubstituted positions (alanine or glycine in the wild-type protein) are blank.

We previously hypothesized that results from the alanine-scanning mutagenesis outcomes might be used as a proxy to identify rheostat positions: if the alanine substitution caused an outcome that was intermediate between that of wild-type and zero function, we reasoned that the position is likely to be a rheostat position (3). However, since the overall, observed range of change differed for each of the five parameters, we could use not a simple fold-change calculation to identify rheostat positions. For example, a 3-fold change in a Q parameter would be considered an intermediate value (and thus indicate a rheostat position), whereas a 3-fold change in a K parameter would not be significant.

Thus, we here normalized the alanine scan calculations, so that outcomes on the five functional parameters can be directly compared. These new results are presented on the PYK-SubstitutionOME supporting worksheet ‘hLPYK Normalized AlaScan Data’; details of these new calculations are in Supplemental Information 3. From these calculations, we next determined the number of parameters (between 0 and 5) with values that were either intermediate between wild type/dead or enhancing. This number is summarized on the ‘Features of PYK Positions’ worksheet in the row ‘Number of param’s in rheostat range’. As observed for positions in and near the allosteric sites (10), substitutions at numerous positions in hLPYK simultaneously modulated multiple functional parameters. Overall, >30% of all hLPYK positions appear to be rheostatic for at least one parameter.

Next, the database includes results for substitutions in hLPYK and ZmPYK. In both isozymes, many positions were assessed with semiSM mutagenesis, so that they could be assigned as having a toggle, rheostat or neutral character. As described in Supplemental Information 1, making these positional classifications requires at least 10–12 substitutions per position. Since putting all data for each variant in the main table would be unwieldy, results for individual variants are included in the supporting worksheets ‘hLPYK Variants’ and ‘ZmPYK Variants’.

To summarize the nature of each substituted position, we used the toggle, neutral and rheostat scores calculated using the RheoScore calculator (5) for each measured parameter. For non-allosteric ZmPYK, for which only *K_a-PEP_* was measured, all three RheoScale scores are reported in the main worksheet for each substituted position. For allosterically regulated hLPYK, five parameters were measured for each variant and thus each position has five sets of three RheoScale scores (15 total), as well as ‘composite neutral’ scores. Composite neutral is the fraction of measured parameters that are like wild type at one position (i.e. all parameters measured for all substitutions at that position) (9). For example, for one position with 10 variants and five parameters, if 48 parameters were like wild type, the score would be 48/50 = 0.96. In the PYK-SubstitutionOME database, the complete scores for each position are reported in the supporting worksheet ‘hLPYK RNT Scores’. Since these tables are so large, summaries—including (i) the maximal rheostat and toggle scores observed among the five parameters and the composite neutral score and (ii) the number of parameters that have scores above the phenotype threshold—as well as overall position assignments are included in the main ‘Features of PYK Positions’ worksheet (rows 45–51). The thresholds used for these assignments are described in Supplemental Information 1 and the worksheet ‘Legends and Definitions’.

The classification of position types provides one example for the utility of integrating many different characteristics of each position. For example, we previously found that rheostat positions are enriched at non-conserved positions that have an evolutionary pattern that follows speciation (9). That is, rheostat positions are conserved in subfamilies but not across the whole family of proteins. In a second example, positions with extreme ‘least patterned’ scores showed a greater propensity for neutral variants (6).

So that all hLPYK variant data can be readily searched as a single dataset, the data from the whole-protein alanine substitution and semiSM studies are also included in the ‘hLPYK Variants’ supporting worksheet, along with results for other published and unpublished hLPYK variants from our laboratory. In total, this dataset comprises more than 1000 biochemically characterized variants. The unpublished variants were generated for a variety of pilot projects and were partially purified/characterized as described by Ishwar *et al.* (25). Notably, several of the unpublished variants are associated with PKD and should be cross-referenced with the ‘Human Disease Mutants’ worksheet.

Finally, we collated a wide range of published biochemical studies performed by us and other labs for variants in PYK isozymes from a variety of species. Given their disparate measurements and isozymes, we include these data in a separate supporting worksheet entitled ‘Other Isozyme Variants’. Within this section, published works are denoted by their PMIDs and each substituted isozyme position was mapped to the analogous position in hLPYK. The Python program and a description of this mapping algorithm are included in Supplemental Information 2. Note that some of these isozymes bind fructose-2,6-bisphosphate, which is designated ‘F26BP’ in the database.

### Structure-based studies

**Figure 6.**
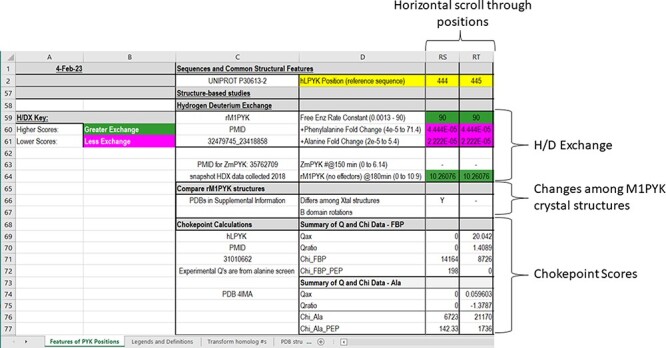
The bottom section of the ‘Features of PYK Positions’ worksheet in the PYK-SubstitutionOME workbook. Again, Rows 1 and 2 and Columns A–D are anchored to provide reference points. This section contains structural information, including H/DX data from both rM1PYK and ZmPYK, structural comparisons for M1PYK and chokepoint calculations for hLPYK. H/DX scores are color-coded to aid visual interpretation; color legends are in the left-most columns.

In the final section of the main worksheet of the PYK-SubstitutionOME workbook (Figure 6), we report results from several studies that leverage various PYK structures. First, we include results for hydrogen/deuterium exchange as detected by mass spectrometry (H/DX-MS) for both rM_1_PYK (61, 62) and ZmPYK (17). These data provide clues about which areas of the protein exchange more and less rapidly (which include information on both conformation and protein dynamics).

**Figure 7.**
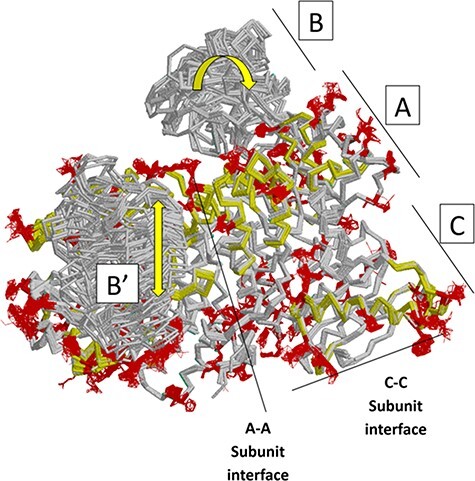
Locations of changes in the domains A and C were detected by comparing monomeric structures of rM1PYK. To highlight the many changes located at the A–A subunit interface, representative dimers were extracted and superimposed from each PDB; note that the perspective of the left-most monomer is looking down at the top of its B domain (B’). The other two monomers of the rM1PYK homotetramer are not shown. The locations of the largest backbone shifts are shown in light yellow (>3.3 Å; most >10 Å); and the locations of the largest side-chain differences are in dark red (>3.3 Å; most >10 Å). Since the entire B domains experience change (yellow arrows), they are not individually highlighted.

Next, we summarize a comparison of 61 subunits from eleven rM_1_PYK structures, which were determined under a variety of liganded states and solution conditions (Figure 7; Supplemental Information 4) and can be compared to the H/DX-MS data. Side chains and backbones that showed >3.3 Å shifts were noted; the majority of the observed changes were >10 Å. Our rationale was that the differences among structures reflect the minimal structural ensemble, which in turn gives information about dynamics of this protein structure. It is now well recognized that protein dynamics contribute to many aspects of protein function, including catalysis, ligand binding and allosteric regulation. More details about these comparisons, along with zoomed-in images of the dynamic regions, are presented in the ‘rM1PYK Structure Comparison’ worksheet in the PYK-SubstitutionOME workbook.

With our rM_1_PYK analysis, we also include three new rM_1_PYK structures that have not previously been published: the novel liganded states are (i) a 2.4-Å structure of the M_1_–PYK–Mg^2+^–Na^+^ complex, (ii) a 2.3-Å structure of the M_1_–PYK–Mg^2+^–pyruvate–K^+^ complex and (iii) a 2.2-Å structure in which the various subunit active sites are populated by either a phosphonate analogue of PEP or succinate in both the presence and absence of adenosine diphosphate (ADP). Experimental details of these new structures are in Supplemental Information 5 and Supplemental Table S1. Importantly, one new structure contained a subunit bound to the phosphonate analogue of both PEP and ADP, which is the best approximation to date of the Michaelis complex for the physiologically relevant PKY reaction in any isoform.

Finally, the last rows within the ‘Structure-Based Studies’ section of the main PYK-SubstitutionOME worksheet summarize results of calculated mechanical coupling (‘Chokepoint calculations’) between active and allosteric sites of hLPYK (108). These chokepoint scores were derived by combining various adjacency matrixes determined using structural and dynamics features of hLPYK (such as Euclidean geometries and covariation in coarse-grained molecular dynamics simulations) to create a final matrix that was then analyzed for the one-site (χ^allosteric site^) and two-site (χ^allosteric site, PEP^) cost-weighted betweenness centrality parameters of the nodes. Large values indicate that a large number of pathways pass through that node, which is in turn hypothesized to identify the pathway by which allosteric communication is transmitted. The measured allosteric couplings for the whole-protein hLPYK alanine scan (‘Q’) were used to benchmark the chokepoint calculation.

## Conclusion

The PYK-SubstitutionOME collection of biochemical characterizations for >1700 PYK substitutions, along with accompanying bioinformatic and structural information, fills a unique information niche. Indeed, very few such datasets include biochemical characterizations for multiple substitutions at many different individual protein positions (i.e. the semiSM design). In our own research, we have already used this database to draw a number of important conclusions about PYK function in particular and protein evolution in general (3, 6, 9, 10, 17, 19, 24–26, 62). We are confident that this database will be equally valuable to other researchers developing methods to predict the outcomes of amino acid substitutions. Without doubt, new measurements, methodologies and data types will be generated in future studies of PYK isozymes. We welcome input from the research community for adding such new information to the PYK-SubstitutionOME database.

## Supplementary Material

baad030_Supp

## Data Availability

The PYK-SubstitutinonOME database is available at https://github.com/djparente/PYK-DB.
